# Epidemiology, species composition and genetic diversity of tetra- and octonucleated *Entamoeba* spp. in different Brazilian biomes

**DOI:** 10.1186/s13071-021-04672-y

**Published:** 2021-03-17

**Authors:** Deiviane Aparecida Calegar, Kerla Joeline Lima Monteiro, Polyanna Araújo Alves Bacelar, Brenda Bulsara Costa Evangelista, Mayron Morais Almeida, Jéssica Pereira dos Santos, Márcio Neves Boia, Beatriz Coronato-Nunes, Lauren Hubert Jaeger, Filipe Anibal Carvalho-Costa

**Affiliations:** 1 Laboratório de Epidemiologia e Sistemática Molecular, Instituto Oswaldo Cruz, Fundação Oswaldo Cruz, Rio de Janeiro, RJ Brazil; 2 Centro/Norte, Escritório Técnico Regional - Fundação Oswaldo Cruz, Piauí, Rua Magalhães Filho, 519, Teresina, Piauí Brazil; 3 Laboratório de Biologia e Parasitologia de Mamíferos Silvestres Reservatórios, Instituto Oswaldo Cruz, Fundação Oswaldo Cruz, Rio de Janeiro, RJ Brazil; 4 Faculdade de Medicina de Petrópolis (FMP)/Faculdade Arthur Sá Earp Neto (FASE), Rua Machado Fagundes, 326, Cascatinha, Petrópolis, Rio de Janeiro Brazil; 5 Faculdade de Farmácia, Universidade Federal de Juiz de Fora, Rua José Lourenço Kelmer, s/n – Campus Universitário, Bairro São Pedro, Juiz de Fora, Minas Gerais Brazil

**Keywords:** *Entamoeba* genus, Molecular epidemiology, Small subunit ribosomal DNA, Intra- and interspecific diversity

## Abstract

**Background:**

*Entamoeba* species harbored by humans have different degrees of pathogenicity. The present study explores the intra- and interspecific diversity, phylogenetic relationships, prevalence and distribution of tetra- and octonucleated cyst-producing *Entamoeba* in different Brazilian regions.

**Methods:**

Cross-sectional studies were performed to collect fecal samples (*n* = 1728) and sociodemographic data in communities located in four Brazilian biomes: Atlantic Forest, Caatinga, Cerrado, and Amazon. Fecal samples were subjected to molecular analysis by partial small subunit ribosomal DNA sequencing (SSU rDNA) and phylogenetic analysis.

**Results:**

Light microscopy analysis revealed that tetranucleated cysts were found in all the studied biomes. The highest positivity rates were observed in the age group 6–10 years (23.21%). For octonucleated cysts, positivity rates ranged from 1 to 55.1%. Sixty SSU rDNA *Entamoeba* sequences were obtained, and four different species were identified: the octonucleated *E. coli*, and the tetranucleated *E. histolytica*, *E. dispar*, and *E. hartmanni*. Novel haplotypes (*n* = 32) were characterized; however, new ribosomal lineages were not identified. The *Entamoeba coli* ST1 subtype predominated in Atlantic Forest and Caatinga, and the ST2 subtype was predominant in the Amazon biome. *E. histolytica* was detected only in the Amazon biome. In phylogenetic trees, sequences were grouped in two groups, the first containing uni- and tetranucleated and the second containing uni- and octonucleated cyst-producing *Entamoeba* species. Molecular diversity indexes revealed a high interspecific diversity for tetra- and octonucleated *Entamoeba* spp. (*H* ± SD = 0.9625 ± 0.0126). The intraspecific diversity varied according to species or subtype: *E. dispar* and *E. histolytica* showed lower diversity than *E. coli* subtypes ST1 and ST2 and *E. hartmanni*.

**Conclusions:**

Tetra- and octonucleated cyst-producing *Entamoeba* are endemic in the studied communities; *E. histolytica* was found in a low proportion and only in the Amazon biome. With regard to *E. coli*, subtype ST2 was predominant in the Amazon biome. The molecular epidemiology of *Entamoeba* spp. is a field to be further explored and provides information with important implications for public health.

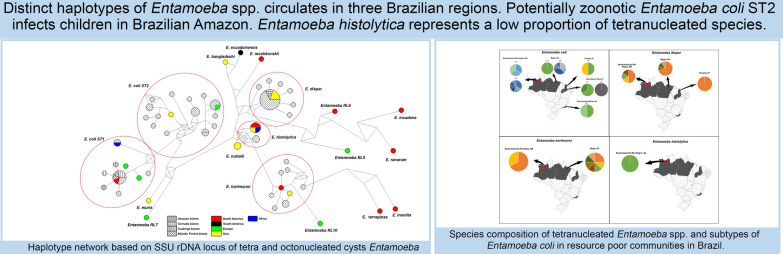

**Supplementary Information:**

The online version contains supplementary material available at 10.1186/s13071-021-04672-y.

## Background

*Entamoeba* species harbored by the human digestive tract have different degrees of pathogenicity and impact on public health [1]. Although some species are considered commensal and non-pathogenic, *E. histolytica* can cause serious, life-threatening, and invasive infections such as amoebic dysentery and liver abscess [2]. *Entamoeba histolytica* produces tetranucleated cysts which are indistinguishable from those produced by *E. dispar*. The similarity of the cysts led to the adoption of the nomenclature *E. histolytica/E. dispar* complex, which also includes *Entamoeba moshkovskii*, another tetranucleated cyst-producing species. Although *E. dispar* and *E. moshkovskii* are considered to have less pathogenic potential [3], they have occasionally been associated with invasive disease [4, 5]. These findings have led to the need for further studies to assess the epidemiology of indistinguishable tetranucleated amoebas [3]. *Entamoeba* species that parasitize other animals can also infect humans [6].

*Entamoeba coli,* which are considered a commensal and harmless organism, produce octonucleated cysts and can be considered a marker of inadequate sanitary conditions, denoting greater exposure to other fecal pathogens. Recently, however, potential pathogenicity has been attributed to this species. Mexican children infected with *E. coli* have higher levels of stool leucocytes than uninfected children, pointing to the possibility of intestinal inflammatory activity triggered by this organism [7, 8]. Additionally, considerable intraspecific genetic variability has been demonstrated for *E. coli*, which can be divided into two subtypes: *E. coli* ST1 and *E. coli* ST2 [9].

Typically, the clinical detection of *Entamoeba* species in fecal samples has been performed through light microscopy. However, the overlap of morphological characteristics between some species and the morphological and size variation in structures are limiting factors for microscopic species-specific diagnosis [10]. The implementation of molecular tools for *Entamoeba* species characterization has enabled a greater understanding of their taxonomy, phylogenetic relationships and epidemiology [11].

Despite recent advances, the proportion of households without sanitation systems in many regions of Brazil remains high. The country has great socioeconomic, environmental and demographic diversity, and appropriate access to drinking water is restricted in many communities. Similarly, peri-urban and urban communities in large Brazilian cities in more industrialized states frequently have poor sanitation infrastructure. In the present study, we explored the species composition, the inter- and intraspecific genetic diversity and phylogenetic relationships, and the prevalence and distribution of *Entamoeba* species infecting populations living in different Brazilian biomes.

## Methods

### Description of study area and population, study design and sampling

**Fig. 1:**
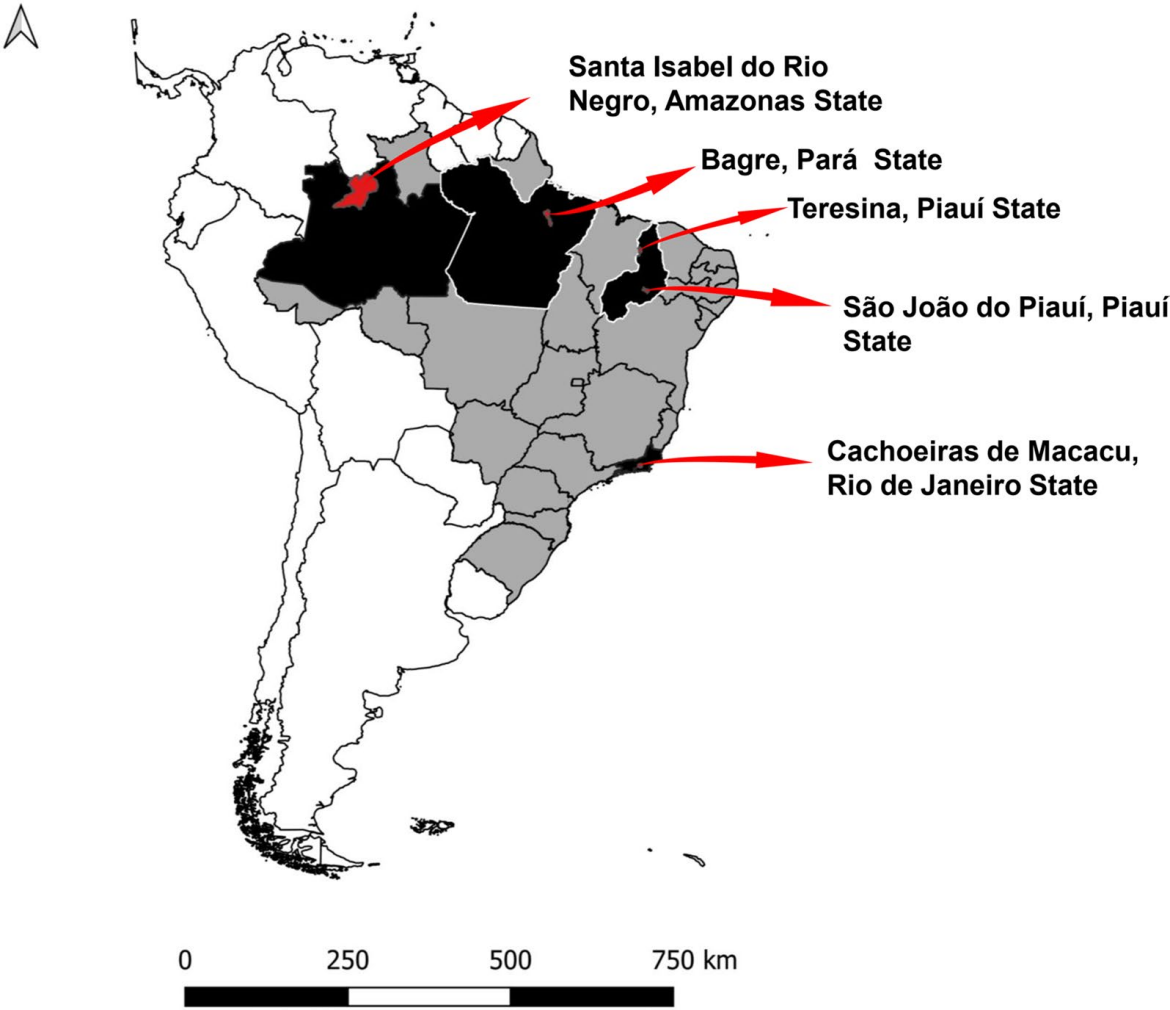
Map of the studied areas in Brazil. The map was created using Google Earth Pro

Communities from cities located in four Brazilian biomes were selected: Cachoeiras de Macacu (CAM) in the state of Rio de Janeiro (Atlantic Forest biome), Teresina (TER) and São João do Piauí (SJPI) in Piauí (Cerrado and Caatinga biomes, respectively), and Santa Isabel do Rio Negro (SIRN) in Amazonas and Bagre (BAG) in Pará (Amazon biome) (Fig. 1). In the five municipalities, the studied communities had precarious access to drinking water and poor sanitation systems, to varying degrees and for distinct reasons. Table 1 shows the socio-environmental and demographic characteristics of each municipality and the number of individuals included in each one. These regions differ with respect to climate, proportion of the population living in poverty and human development index, among other parameters. Cross-sectional studies were carried out in the included areas to collect fecal samples and sociodemographic data. *Entamoeba* spp. fecal samples were identified as positive using parasitological methods (Ritchie’s modified ethyl acetate centrifugation) [12].

**Table 1:** Sampling, sociodemographic and environmental characteristics of distinct study areas

Characteristic	Biome				
	Amazon		Cerrado and Caatinga		Atlantic forest
	Santa Isabel do Rio Negro (AM)	Bagre (PA)	Teresina (PI)	São João do Piauí (PI)	Cachoeiras de Macacu (RJ)
Population	22.404	30.673	864.845	20.601	58.937
Human development index	0.479	0.471	0.751	0.645	0.700
Gini index	0.35	0.37	0.50	0.45	0.45
Year of study	2011	2020	2017	2018	2018
Localization of districts included	Urban	Urban	Rural	Rural	Urban and rural
Fecal samples	392	362	298	131	545
Income (MPCHI*, USD**)					
≤ 45	302 (77%)	209 (57.7%)	153 (51.4%)	67 (51.1%)	192 (35.2%)
> 45	90 (23%)	163 (44.9%)	145 (48.6%)	64 (48.9%)	290 (53.2%)
Data not available	–	–	–	–	63 (11.6%)
Water supply	Negro River and artesian wells	Furo Santa Maria River (Baía de Marajó)	Artesian wells	Artesian wells and water stored in cistern	Macacu River and artesian wells
% open evacuation	–	142/362 (39%)	106/298 (35.5%)	63/131 (48%)	–
Gender					
Male	207	188	147	69	284
Female	185	174	151	62	261
Age group (years)					
0–2	87	80	11	7	99
3–5	108	83	15	7	120
6–10	142	135	29	14	184
11–15	49	62	35	27	235
> 15	–	–	207	76	5
Data not available	6	–	1	–	43

*AM* Amazonas, *PA* Pará, *PI* Piauí, *RJ* Rio de Janeiro

*MPCHI—monthly per capita house income, **USD 1 = BRL 4

### DNA extraction, polymerase chain reaction (PCR) and DNA sequencing

Fecal samples positive on light microscopy were subjected to molecular analyses for species characterization and phylogenetic studies. In addition, some *Entamoeba* spp.-negative samples on microscopy were selected, randomly or when another individual from the same household was positive, to be tested by PCR. This was performed in order to improve the number of DNA sequences for genetic analyses. Genomic DNA was extracted from 200 µl of the sedimented fecal material using the ZR Fungal/Bacterial DNA MiniPrep™ extraction kit (Zymo Research, Irvine, CA, USA). PCR was performed using the Platinum Taq DNA Polymerase kit (Invitrogen, Waltham, MA, USA), with a final volume of 50 μl. The small subunit rRNA gene locus (SSU rDNA) of *Entamoeba* spp. (550 base pairs [bp]) was targeted for amplification, as described in Verweij et al. [13]. Amplification conditions were as follows: each deoxynucleoside triphosphate at 200 mM, 25 pmol of each specific primer, 10X PCR buffer, 1 U of Taq DNA Polymerase, and 5 μl of the DNA sample. The thermal cycling conditions were as follows: initial denaturation of 5 min at 94 °C, 35 cycles of 30 s at 94 °C, 30 s at 55 °C, and 30 s at 72 °C; and final extension of 2 min at 72 °C. The PCR products were purified with polyethylene glycol (PEG) [14] and sequenced using the BigDye Terminator v3.1 kit (Applied Biosystems, Foster City, CA, USA) in an ABI 3730 automated DNA sequencer (Applied Biosystems).

### Data analysis

The obtained sequences were edited and analyzed using BioEdit version 7.2.5 software [15]. The Basic Local Alignment Search Tool (BLASTn; NCBI https://www.ncbi.nlm.nih.gov/) was used to verify similarity with *Entamoeba* species. The obtained sequences were deposited in GenBank under accession numbers MW026735–MW026794. BioEdit version 7.2.5 [15] was used to align and cut the sequences into equal fragments (542 bp). Phylogenetic inferences were performed using Molecular Evolutionary Genetics Analysis (MEGA) version 7.0.20 software [16]. The maximum likelihood (ML) and neighbor joining (NJ) methods were applied. The substitution model for the data set was chosen using the Bayesian information criterion (BIC) in MEGA version 7.0.20 software [16]. According to the lower BIC score, the Tamura 3-parameter model (T92) was chosen. Branch support was provided by bootstrapping with 1000 replications. *Entamoeba* spp. orthologous sequences (*n* = 46) were used to construct an alignment using the BLASTn tool against the Nucleotide Collection (nr/nt) database (https://www.ncbi.nlm.nih.gov/nucleotide/) (Additional file 1: Table S1). The reference sequences were selected to be representative of the genus. Sequences with degenerate bases were not included.

A median-joining (MJ) haplotype network was constructed in Network version 10.1.0.0 software [17] (www.fluxusengineering.com), with the input file previously prepared in DnaSP (DNA Sequence Polymorphism) version 6 software [18]. Diversity indexes of tetra- and octonucleated *Entamoeba* populations were determined using the pairwise distance in Arlequin version 3.5.2.2 software [19]. The pairwise F_st_ (fixation index) value was tested in all populations using Arlequin version 3.5.2.2 software [19] to estimate the extent of genetic differentiation among populations with a significance of 1000 random permutations.

## Results

### Study population

**Fig. 2:**
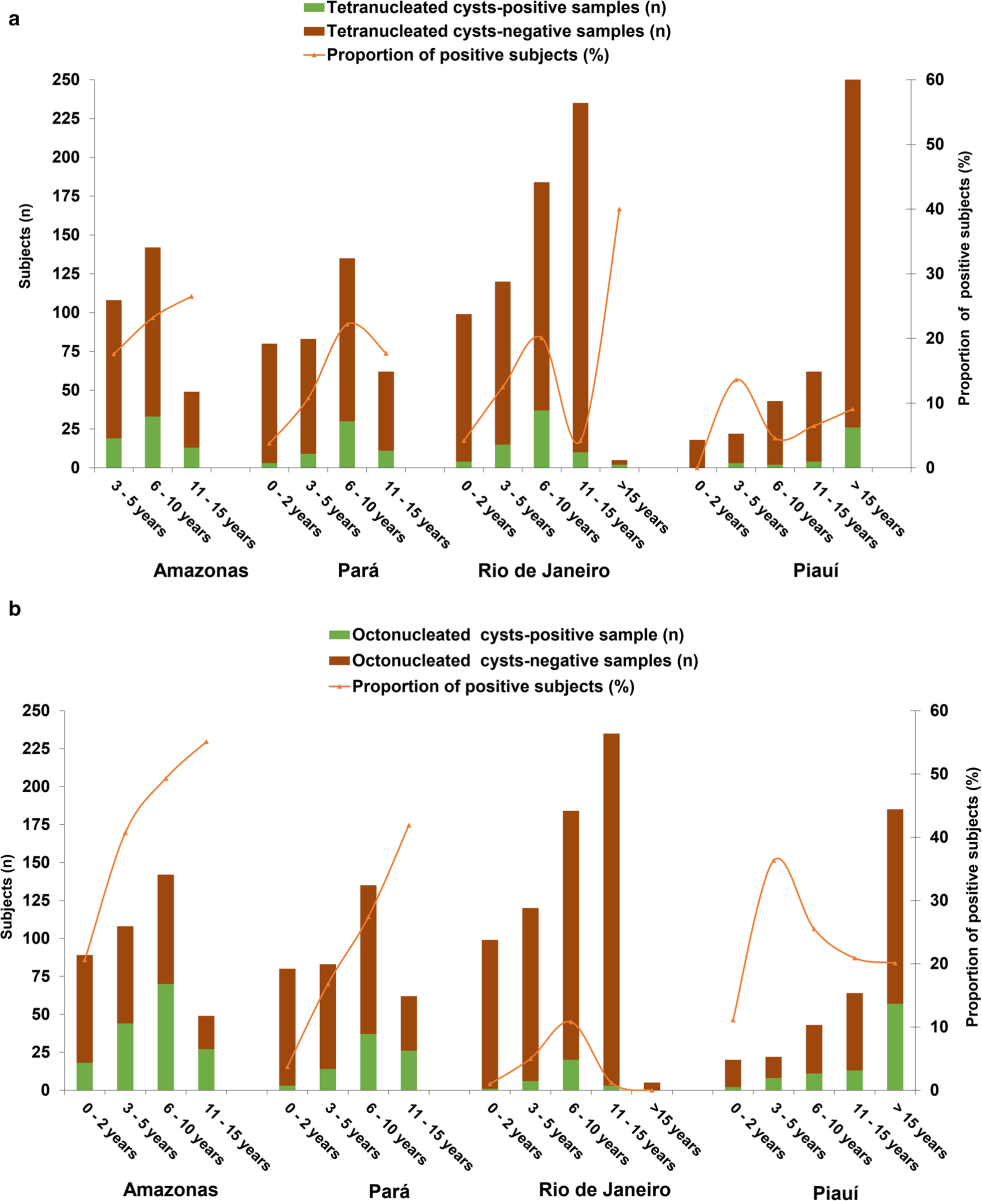
The green and brown bars show the number of positive and negative fecal samples for the presence of tetranucleated (**a**) and octonucleated (**b**) *Entamoeba* cysts by light microscopy among subjects studied, by age group, in the four states; red lines depict the proportion of positive samples

Figure 2 presents the detection rates for tetra- and octonucleated *Entamoeba* cysts in fecal microscopic examinations in distinct age groups and settings. In the Amazon and Atlantic Forest biomes, tetranucleated cysts were found in fecal samples from children up to 2 years of age, with positivity rates ranging from 3.8 to 10.3%, respectively. In these biomes, one of the highest rates was found in the age group of 11–15 years, reaching 26.5%. In the four studied states, octonucleated cysts were detected through microscopy in 1–20.6% of children up to 2 years of age, and 10.8–49.3% of children aged 6–10 years.

### Molecular epidemiology and genetic diversity of Entamoeba spp.

**Fig. 3:**
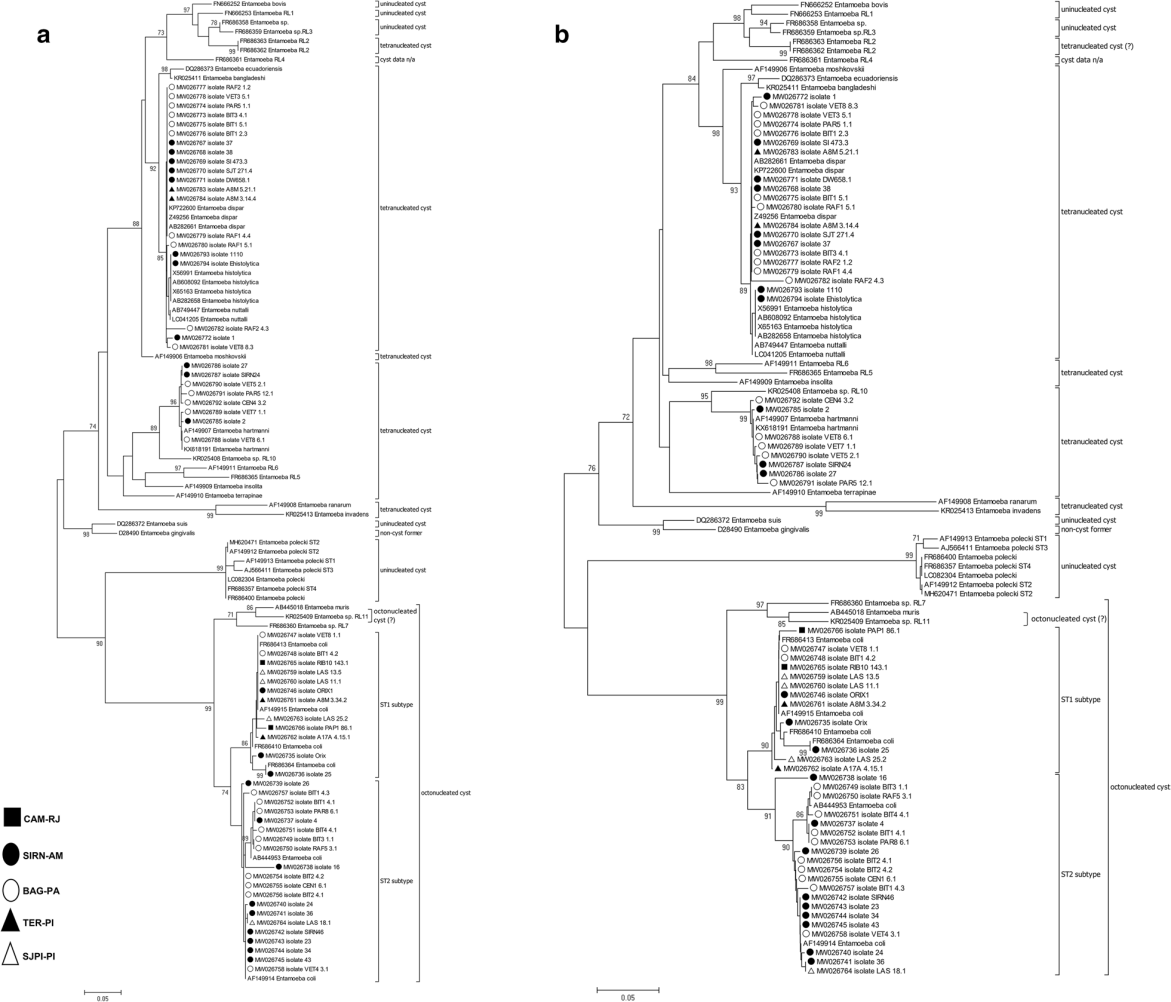
**a** Maximum likelihood and **b** neighbor-joining trees inferred from uni-, tetra- and octonucleated cyst-producing *Entamoeba* spp. SSU rDNA locus (542 bp, *n* = 106). Support for the branching order was determined b 1,000 bootstrap replicates, and only values > 70% are reported. The number of nuclei in the mature cyst are shown. *BAG* Bagre, *CAM* Cachoeiras de Macacu, *SIRN* Santa Isabel do Rio Negro, *SJPI* São João do Piauí, *TER* Teresina. GenBank accession numbers are indicated. Further details for reference strains can be found in Additional file 1: Table S1

A total of 60 SSU rDNA *Entamoeba* spp. sequences (542 bp) were obtained from fecal samples. The BLAST analyses revealed four different *Entamoeba* species: *E. coli* (*n* = 32), *E. dispar* (*n* = 18), *E. hartmanni* (*n* = 8) and *E. histolytica* (*n* = 2) (Table 2). With regard to the *E. histolytica*/*E. dispar* complex, the Amazon biome presented the greatest species diversity. In Piauí, only *E. dispar* was identified. With respect to *E. coli*, ST1 predominated in Rio de Janeiro and Piauí, and ST2 was predominant in Amazonian states (Amazonas and Pará). Figure 3 displays the ML and NJ trees inferred from uni-, tetra- and octonucleated *Entamoeba* spp. cysts, with a total of 106 *Entamoeba* SSU rDNA sequences (*n* = 60 sequences from the present study plus 46 reference sequences). Two major groups were observed, the first one containing uni- and tetranucleated cyst-producing *Entamoeba* species, and the second containing *Entamoeba* species producing uni- and octonucleated cysts. The main differences between the two trees were as follows: (i) in the ML tree, *E. moshkovskii* was grouped in a single clade (Fig. 3a), while in the NJ tree it was grouped together with other tetranucleated cyst-producing Entamoeba species (Fig. 3b); and (ii) *Entamoeba* RL5, RL6 and *E. insolita* were grouped in a single clade on the NJ tree, while on the ML tree they were grouped close to *E. hartmanni*.

**Table 2:** Molecular diversity indexes of tetra- and octonucleated cyst-producing *Entamoeba* based on SSU rDNA locus (542 bp, *n* = 89)

Species (*N*)	Region (*N*)	Statistics					
		*H* ± SD	No. of haplotypes	No. of polymorphic sites	No. of substitutions	No. of transitions	No. of transversions
*E. coli* (38)	All ST1 (16)	0.816 ± 0.095	9	45	44	27	17
	Brazil (12)	0.772 ± 0.127	7	44	44	27	17
	BAG + SIRN (5)	0.700 ± 0.218	3	5	5	3	2
	TER (2)	1.0000 ± 0.5000	2	1	1	1	0
	SJPI (3)	0.6667 ± 0.3143	2	4	4	2	2
	CAM (2)	1.000 ± 0.500	2	7	7	3	4
	All ST2 (22)	0.952 ± 0.029	15	52	57	26	31
	Brazil (20)	0.957 ± 0.028	14	52	57	26	31
	BAG + SIRN (19)	0.959 ± 0.030	14	52	57	26	31
*Entamoeba dispar* (21)	All (21)	0.500 ± 0.132	7	26	22	16	6
	Asia (3)	0.000 ± 0.000	1	0	0	0	0
	Brazil (18)	0.568 ± 0.137	7	26	22	16	6
	TER (2)	0.625 ± 0.139	7	26	22	16	6
	BAG + SIRN (16)	0.000 ± 0.000	1	0	0	0	0
*E. hartmanni* (10)	All (10)	0.977 ± 0.054	9	28	24	16	8
	Brazil (8)	0.964 ± 0.077	7	28	24	16	8
*E. histolytica* (6)	All (6)	0.533 ± 0.172	2	4	2	1	1
	North America (3)	0.000 ± 0.000	1	0	0	0	0
	Brazil (2)	0.000 ± 0.000	1	0	0	0	0
All* (89)		0.9625 ± 0.0126	55	353	272	203	199

*H* ± SD: gene diversity ± standard deviation. All*: *E. dispar*, *E. histolytica*, *E. hartmanni*, *E. coli*, *Entamoeba *sp., *E. moshkovskii*, *E. ecuadoriensis*, *E. bangladeshi*, *E. nuttalli*, *E. muris*, *E. terrapinae*, *E. insolita*, *E. invadens*, *Entamoeba* RL7, *Entamoeba* RL10*, Entamoeba* RL5, *Entamoeba* RL6. Further details for reference strains can be found in Additional file 1: Table S1. Bold indicates sequences obtained in this study (Brazil). *BAG* Bagre, *CAM* Cachoeiras de Macacu, *SIRN* Santa Isabel do Rio Negro, *SJPI* São João do Piauí, *TER* Teresina

Among 28 samples containing tetranucleated *Entamoeba* cysts, only two were characterized as the pathogenic *E. histolytica*. The other 26 were identified as *E. hartmanni* or *E. dispar*. *Entamoeba histolytica* was identified only among isolates from the Amazon biome (Amazonas, *n* = 2); *E. dispar* was identified in the Cerrado (Teresina, Piaui, *n* = 2) and Amazon biomes (Pará, *n* = 10, and Amazonas, *n* = 6). *E. hartmanni* was characterized only in the Amazon biome (Pará and Amazonas in five and three samples, respectively). No *Entamoeba moshkovskii* or uninucleated cyst-producing *Entamoeba* species were found.

**Fig. 4:**
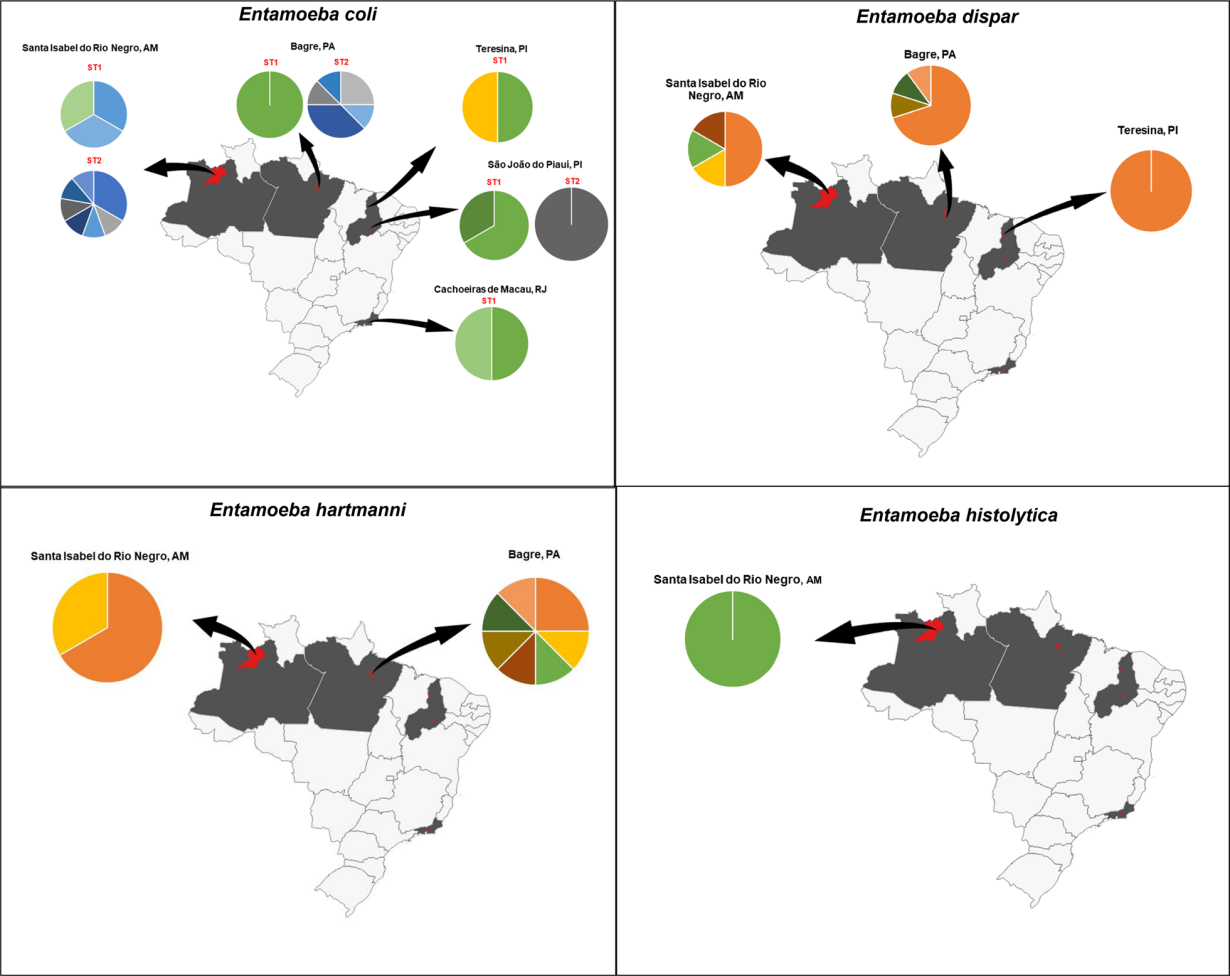
Map of the haplotypes found by locality, Brazil. The colors in the graphs represent the diversity of haplotypes found for each species. The map was created using QGIS version 3.12.3

*Entamoeba coli* was the species most commonly found (*n* = 32) and was present in all localities studied. Both *E. coli* subtypes ST1 (*n* = 12; 37.5%) and ST2 (*n* = 20; 62.5%) were identified. Subtype ST1 was described in all regions studied (Piauí, *n* = 5; Rio de Janeiro, *n* = 2; Amazonas, *n* = 3; and Pará, *n* = 2), while ST2 was found almost exclusively in the Amazon region (Amazonas, *n* = 9 and Pará, *n* = 10) (Fig. 4).

**Fig. 5:**
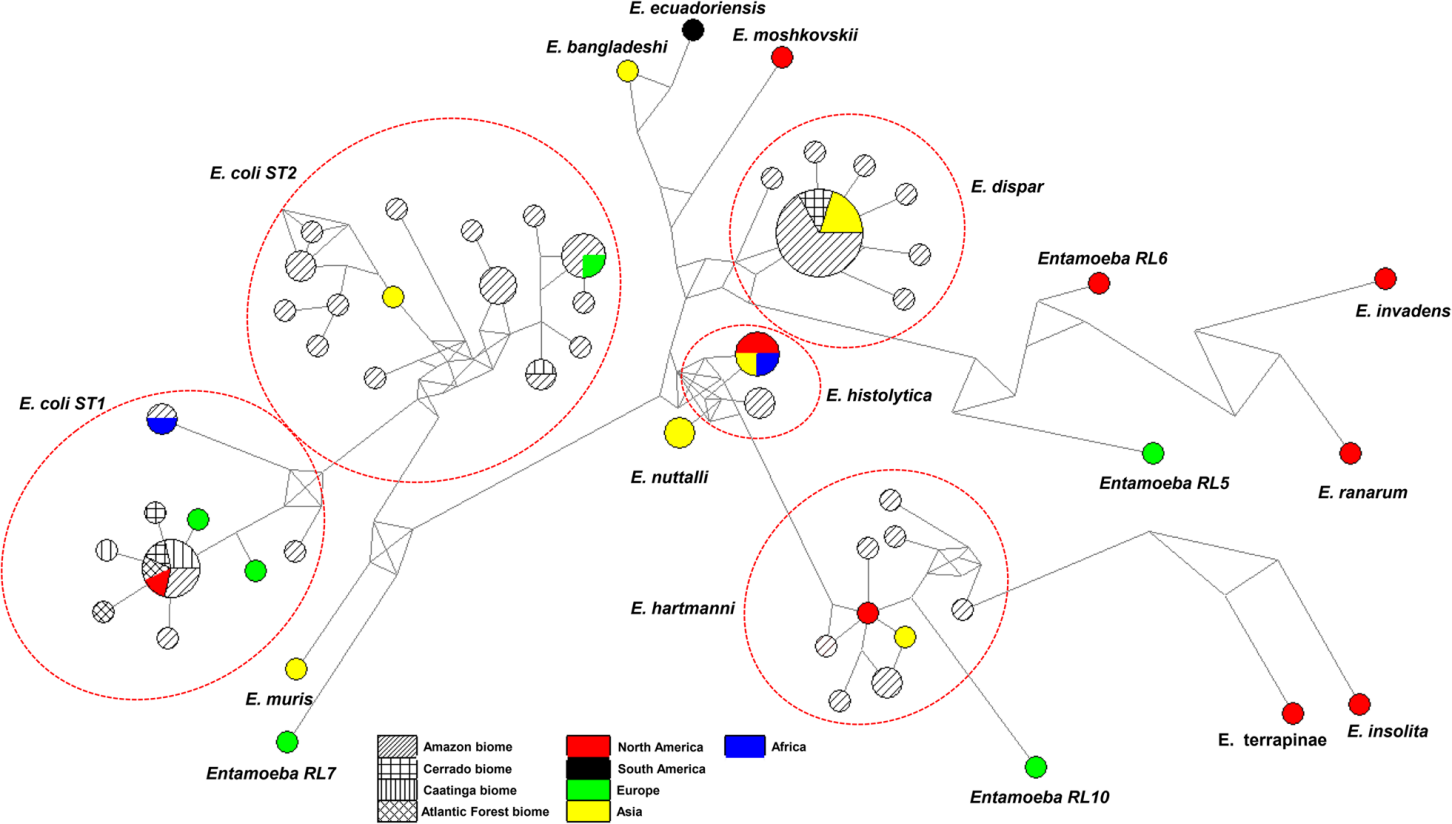
Haplotype network based on SSU rDNA locus of tetra- and octonucleated cysts of *Entamoeba* (542 bp, *n* = 89). Area of the circle is proportional to number of sequences

The MJ haplotype network based on tetra- and octonucleated cyst-producing *Entamoeba* showed a similar topology to the ML tree discriminating species or subtypes (Fig. 5). A total of 60 SSU rDNA sequences from the present study plus 29 reference sequences were distributed in 55 haplotypes. Thirty-six different haplotypes were identified in our sequences, and several new haplotypes were found. In *E. coli* ST1 and ST2, five and 13 new haplotypes were characterized, respectively. *Entamoeba hartmanni, E. dispar* and *E. histolytica* had seven, six and one new haplotype, respectively. *Entamoeba dispar* and *E. coli* subtype ST1 presented a star-shaped haplotype network (Fig. 5), with a central and dominant haplotype. Interestingly, most of our samples belong to this ancestral haplotype.

Analysis of molecular diversity indexes revealed high interspecific diversity for tetra- and octonucleated *Entamoeba* spp., with *H* ± SD = 0.9625 ± 0.0126 and 353 polymorphic sites (Table 2). The intraspecific diversity varied according to species or subtype. *Entamoeba dispar* and *E. histolytica* showed lower intraspecific variability (*H* ± SD = 0.500 ± 0.132 and 0.533 ± 0.172, respectively) than *E. coli* subtypes ST1 and ST2 and *E. hartmanni* (*H* ± SD = 0.816 ± 0.095, 0.952 ± 0.029, and 0.977 ± 0.054, respectively) (Table 2). The intraspecific variability of these two species was similar to the interspecific variability for the species. The F_st_ results corroborate the diversity analysis results (Additional file 2: Table S2). The *E. dispar* sequences from different Brazilian biomes showed low F_st_ values, indicating no isolation between populations. For *E. coli* ST1 and ST2 sequences, even with high genetic variability, there was no evidence of significant isolation between populations (Additional file 2: Table S2 and Fig. 5).

Tetra- and octonucleated cyst co-infections were identified by microscopy in 18.6% (72/387) of fecal samples. Of these, 39 samples were subjected to PCR. Overlapping peaks were not observed in the sequences, indicating failure to detect co-infections. Table 3 shows the results obtained by microscopy and PCR. All fecal samples collected (*n* = 1728) were subjected to the traditional parasitological technique (Ritchie’s modified ethyl acetate centrifugation) and microscopy. It was not possible to perform PCR on all samples due to technical or logistical limitations. Two hundred and fifty-three samples were subjected to PCR. Of these, 65.3% (77/118) were positive for both techniques and 34.7% (41/118) were PCR-positive and microscopy-negative (Table 3). Finally, 60 *Entamoeba* spp. sequences were successfully obtained (50.8% of PCR amplifications), and many sequences from other organisms were identified, including fungi, bacteria, plants and other intestinal protozoa (*Endolimax*, *Iodamoeba* and *Blastocystis* genera) (data not shown).

**Table 3:** *Entamoeba* spp. microscopy and PCR results in the present study

Biome/locality	Samples	Result				
		Microscopy-positive *N* (%)	PCR-positive* N (%)	Microscopy-positive + PCR-positive *N* (%)	Microscopy-negative + PCR-positive *N* (%)	*Entamoeba* spp. sequences obtained** N (%)
Amazon						
Santa Isabel do Rio Negro (AM)	392	160/392 (40.8)	38/98 (38.7)	16/38 (42.1)	22/38 (57.9)	23/38 (60.5)
Bagre (PA)	362	65/362 (17.9)	36/59 (61)	30/36 (83.3)	6/36 (16.7)	27/36 (75)
Caatinga and Cerrado						
Teresina (PI)	298	59/298 (19.7)	14/26 (53.8)	10/14 (71.4)	4/14 (28.6)	4/14 (28.5)
São João do Piauí (PI)	131	31/131(23.6)	16/33 (48.4)	13/16 (81.3)	3/16 (18.7)	4/16 (25)
Atlantic Forest						
Cachoeiras de Macacu (RJ)	545	72/545 (13.2)	14/37 (37.8)	8/14 (57.1)	6/14 (42.9)	2/14 (14.2)
Total	1728	387/1728 (22.3)	118/253 (46.6)	77/118 (65.3)	41/118 (34.7)	60/118 (50.8)

Only fecal samples positive on light microscopy were subjected to molecular analyses for species characterization and phylogenetic studies. Some *Entamoeba* spp. negative samples on microscopy were selected, randomly or when another individual from the same household was positive, to be tested on PCR. *AM* Amazonas, *PA* Pará, *PI* Piauí, *RJ* Rio de Janeiro. *N*: absolute number. (%): percentage. *Includes microscopy positive and negative results. **Sequences from other organisms were also obtained, including fungi, bacteria, plants and other intestinal protozoa (*Endolimax* genus)

## Discussion

In the present study, we describe high positivity rates for infection by *Entamoeba* spp. in economically vulnerable communities with poor health infrastructure in different regions of Brazil. Amebiasis is a water- and food-borne disease with strong socio-environmental determinants, and its distribution is poverty-related and heterogeneous among human populations [20–22] and remains endemic in many Brazilian regions.

We characterized species and subtypes of *Entamoeba* spp. circulating in different communities. The interspecific variability of *Entamoeba* spp. based in SSU rDNA locus was high, H close to 1. Our results draw attention to the characterization of new haplotypes for all *Entamoeba* species. Of the 36 haplotypes found, only four had already been described. These four haplotypes were in a central region of the haplotype network, indicating their ancestral nature, as in *E. dispar* and in *E. coli* ST1 and ST2. In addition, 14 new haplotypes described here originated from these ancestral haplotypes. Despite this, no new ribosomal lineages were identified. Among the members of the family Entamoebidae, the genus *Entamoeba* is the most well studied due to the pathogenic potential of certain species. In the phylogenetic trees generated in the present study, sequences were grouped into two groups for the *Entamoeba* genus, as in Stensvold et al. [9] and Jacob et al. [23]. However, some differences could be seen. Another limitation of the study is the fact that we obtained sequences of 542 bp, which corresponds to one third the size of the gene chosen for the analyses. This generated relatively low bootstrap values in some branches of the phylogenetic trees.

The most evident difference was that the uninucleated *E. polecki* species was grouped in the same group as the octonucleated *E. coli*, unlike Stensvold et al. [9], where it was grouped with the uni- and tetranucleated species. Our result is similar to the study by Jacob et al. [23], in which *E. polecki* shared a common ancestor with the octonucleated *E. coli*. Another evident difference was *E. moshkovskii,* which shared a common ancestor with uni- and tetranucleated in our ML tree. In contrast, in the NJ tree, this species shared a common ancestor with the tetranucleated species *E. ecuadoriensis*, *E. dispar*, *E. histolytica*, *E. nuttalli* and *E. bangladeshi*, as in Stensvold et al. [9] and Jacob et al. [23]. Additionally, *E. hartmanni* shared a common ancestor with *E. insolita*, *E. terrapinae* and *Entamoeba* RL5 and RL6 in the ML tree. In the NJ tree this species shared a common ancestor with *E. terrapinae*, as seen in Stensvold et al. [9]. Other small differences were seen when compared with Stensvold et al. [9] and Jacob et al. [23]. The differences observed between our study and previous studies can be explained by the fact that we did not use all the sequences available in GenBank, since we selected reference sequences without degenerate bases. Moreover, these results make us wonder whether the SSU rDNA locus or the “number of nuclei in the mature cyst” morphological character are suitable for the taxonomic classification of the *Entamoeba* genus. Apparently much still needs to be studied to understand the genetic complexity of members of this genus.

The main finding regarding tetranucleated cyst-producing *Entamoeba* species was the low proportion of *E. histolytica* found in the obtained sequences. Most sequences were characterized as *E. dispar* or *E. hartmanni*, considered nonpathogenic species. It is speculated that *E. dispar* and *E. hartmanni* are responsible for most infections that were previously considered to be associated with *E. histolytica* [3, 24, 25]. In fact, molecular epidemiological studies show that *E. dispar* is the species most commonly found among the tetranucleated cysts [21, 26–29]. Our results revealed that *E. dispar* presented a wider geographic distribution, whereas *E. histolytica* was identified only in the Amazon biome. Our results corroborate other studies conducted in Brazil, which have suggested that *E. histolytica* is more common in northern and northeastern Brazil and is less frequently detected in other regions [26, 30, 31]. However, we cannot rule out a sample bias and limitations in the PCR technique that could favor the amplification of one species over another.

In this study, we found only one haplotype for *E. histolytica*, and it was distinguished from the haplotype previously described in North America, Africa and Asia (X65163, *E. histolytica* HM-1:IMSS strain) [32–34]. In an overview of the diversity of *Entamoeba histolytica* by Zermeño and colleagues [35], they argue that although many haplotypes are found in only a single country, there are no lineages within the networks that may be related to a particular geographic region or infection outcome. These positive subjects in the present study had no symptoms. We must consider that most *E. histolytica* infections can be asymptomatic and that only 10% of those infected have symptoms [36–38].

The intraspecific variability of *E. hartmanni* was as high as the interspecific variability of the *Entamoeba* genus. In addition, among the eight sequences obtained from *E. hartmanni*, seven different haplotypes were found. Moreover, and all sequences were obtained in the Amazon biome. Only one haplotype obtained has been previously described in humans and nonhuman primates (100% similarity to KX618191 and AF149907, respectively) [39, 40]. Previous studies have suggested low variability for *E. hartmanni* based on restriction fragment length polymorphism and SSU rDNA sequencing [9, 41]. However, the real epidemiological significance of the high diversity found in the present study remains to be clarified.

The phylogenetic trees and MJ network revealed two main clusters for *E. coli* corresponding to the previously described subtypes ST1 and ST2 in Stensvold and colleagues [9]. In the present study, *Entamoeba coli* ST1 had a wider geographic distribution, being identified in all studied biomes, with the presence of five new haplotypes. Two previously described haplotypes were found, one isolated from humans in Nigeria (FR686364) [9] and the other from humans in the USA and nonhuman primates in Germany (AF149915 and FR686410, respectively) [9, 39]. *Entamoeba coli* ST2 subtype was identified in the Caatinga and Amazon biomes, being the predominant subtype in the Amazon. Twenty sequences were obtained and 13 new haplotypes were described. Only one haplotype has been previously described, isolated from humans in England (AF149914) [41]. It is hypothesized that the ST1 subtype is more common than ST2 in humans [42], and ST2 was recently identified in wild nonhuman primates in Asia and Africa [43, 44]. It is intriguing to observe a large number of new *E. coli* ST2 subtypes in humans living in the Amazon rainforest, the habitat of great diversity of neotropical primates.

We observed that 65.3% of the samples had a positive result with both techniques used (microscopy and PCR). Moreover, despite a reasonable number of PCR-positive samples (*n* = 118), we were only successful in sequencing and obtaining *Entamoeba* spp. SSU rDNA sequences in 50.8% of them. This makes us reflect on the limitations of the techniques used as well as of the fecal samples. Many factors can influence the success of the analysis, including low parasitic load in the sample and randomness in pipetting, unspecific PCR amplifications (multiple bands on the agarose gel), limitations in the PCR technique (primer binding, reaction), intrinsic limitations of the fecal samples (presence of microbiota and in different counts), and differences in the sensitivity of the techniques used. In addition, non-*Entamoeba* spp. sequences were obtained in this study, such as fungi or bacteria, which makes the analysis difficult and time-consuming.

The microscopy identified co-infections with octo- and tetranucleated cysts. In contrast, by direct nucleotide sequencing it was not possible to verify the presence of more than one species, and only a small amount of “background” was present in chromatograms. The species identified in the sequencing were related to the number of cysts observed under microscopy, in which more cysts represent more DNA available for PCR. The morphological similarity between *E. histolytica* and *E. dispar*, and the unusual large cysts of *E. hartmanni*, can also lead to misidentification [26], making microscopic analysis problematic for the diagnosis of non-symptomatic amebiasis.

As mentioned above, the communities included in the present study are situated in four different biomes with very distinct climates. Human populations in the Amazon, Caatinga and Cerrado regions have a higher prevalence of intestinal parasites, a higher incidence of diarrheal diseases and a lower proportion of the population with access to adequate sanitation and safe drinking water [45–48]. Although there are great differences in both the supply of drinking water and the water resource management strategies in these regions, in both there is a shortage of clean water for human consumption [47, 49].

In Brazil, intestinal parasitism control strategies target soil-transmitted helminths through mass albendazole administration. Moreover, the precarious conditions of sanitation and water management systems in some regions contributes to the current trend in the etiological profile of intestinal parasitism, characterized by the permanence of protozoan infections, with a reduction in the prevalence of soil-transmitted helminths [50–52]. Even though intestinal infections with soil-transmitted helminths are a major problem, affecting mainly children worldwide, estimates from the Global Burden of Disease Study indicate that other agents, including intestinal protozoa, are responsible for more than 6 million disability-adjusted life years (DALYs) [51].

## Conclusion

Tetra- and octonucleated cyst-producing *Entamoeba* species are endemic in the studied communities, which represent low-income regions with nonexistent or insufficient sanitation systems. The pathogenic *E. histolytica* was found in a low proportion and only in the Amazon biome. Additionally, other tetranucleated species were commonly found in the studied regions. The distribution of *Entamoeba* species in Brazil is clinically important information, since many *E. histolytica*-positive parasitological examinations in fact represent infections with non-pathogenic species within the *E. histolytica/E. dispar* complex or *E. hartmanni*. *Entamoeba coli* subtypes present a geographically uneven distribution, with the ST2 subtype—commonly found in nonhuman primates—being predominant in the Amazon biome.

## Supplementary Information

**Additional file 1: Table S1.** *Entamoeba* spp. reference strains used in the present study.

**Additional file 2: Table S 2.** Population pairwise Fst values based on SSU rDNA locus of tetra- and octonucleated cysts *Entamoeba* (542 bp, *n* = 89).

## Data Availability

The datasets analyzed during the present study are available from the corresponding author upon reasonable request. All sequence files are available from the GenBank database under accession numbers MW026735–MW026794.

## Abbreviations

ST1: Subtype 1
ST2: Subtype 2
CAM: Cachoeiras de Macacu
TER: Teresina
SJPI: São João do Piauí
SIRN: Santa Isabel do Rio Negro
BAG: Bagre
PCR: Polymerase chain reaction
SSU rDNA: Small subunit rRNA gene
ML: Maximum likelihood
NJ: Neighbor-joining
BIC: Bayesian information criterion
MJ: Median joining
