# Supplementary material for: Epidemiology, species composition and genetic diversity of tetra- and octonucleated Entamoeba spp. in different Brazilian biomes
Source: Parasit Vectors. 2021 Mar 17;14:160. doi: 10.1186/s13071-021-04672-y (PMC7968159; doi:10.1186/s13071-021-04672-y)
Supplement: Supplementary file 1 — Additional file 1: Table S1. Entamoeba spp. reference strains used in the present study. [file 13071_2021_4672_MOESM1_ESM.docx]

**Additional file 1: Table S1** *Entamoeba* spp. reference strains used in this study.

| **Mature cyst** | **Species** | **GenBank accession number** | **Isolate** | **Country** | **Continent** | **Host** | **Year** | **Reference** |
| --- | --- | --- | --- | --- | --- | --- | --- | --- |
| Octonucleated | *E. coli* ST1 | FR686413 | EM049 | Sweden | Europe | human | 2010 | [1] |
|  | *E. coli* ST1 | FR686410 | Drill1 | Germany | Europe | monkey | 2010 | [1] |
|  | *E. coli* ST2 | AF149914 | IH:96/135 | England | Europe | human | 1997 | [2] |
|  | *E. coli* ST1 | AF149915 | HU-1:CDC | USA | North America | human | 1999 | [2] |
|  | *E. coli* ST1 | FR686364 | S2702 | Nigeria | Africa | human | 2015 | [1] |
|  | *E. coli* ST2 | AB444953 | UZG-EC-01 | Japan | Asia | monkey | 2007 | [3] |
|  | *E. muris* | AB445018 | MG-EM-01 | Japan | Asia | gerbil | 2007 | [3] |
|  | *Entamoeba* RL7 | FR686360 | 09/1246 | UK | Europe | monkey | 2011 | [1] |
| Tetranucleated | *E. bangladeshi* | KR025411 | 8111 | Bangladesh | Asia | human | 2012 | [4] |
|  | *E. dispar* | AB282661 | SAW1734RclAR | Nepal | Asia | monkey | 2005 | [5] |
|  | *E. dispar* | Z49256 | N/A | India | Asia | human | 1995 | [6] |
|  | *E. dispar* | KP722600 | ED_IQ5 | Iraq | Asia | human | 2013 | [7] |
|  | *E. hartmanni* | AF149907 | 162-2005 | USA | North America | monkey | 1999 | [2] |
|  | *E. hartmanni* | KX618191 | N/A | Singapore | Asia | human | 2016 | [8] |
|  | *E. histolytica* | AB608092 | BF-841 cl1 | Burkina Faso | Africa | human | 2008 | [9] |
|  | *E. histolytica* | AB282658 | HK-9 | Korea | Asia | human | 1991 | [5] |
|  | *E. histolytica* | X65163 | HM 1-IMSS | Mexico | North America | human | 1971 | [10] |
|  | *E. histolytica* | X56991 | HM-1:IMSS | Mexico | North America | human | 1967 | [11] |
|  | *E. insolita* | AF149909 | NIH:1192:1 | USA | North America | turtle | 1995 | [2] |
|  | *E. invadens* | KR025413 | VK-1 | USA | North America | lizard | 2015 | [4] |
|  | *E. moshkovskii* | AF149906 | Laredo | USA | North America | human | 1956 | [2] |
|  | *E. nuttalli* | LC041205 | EM50 | China | Asia | monkey | 2014 | [12] |
|  | *E. nuttalli* | AB749447 | GY4 | China | Asia | monkey | 2012 | [13] |
|  | *E. ranarum* | AF149908 | NIH:1092:1 | USA | North America | frog | 1995 | [2] |
|  | *E. ecuadoriensis* | DQ286373 | EC | Ecuador | South America | sewage | 1964 | [15] |
|  | *E. terrapinae* | AF149910 | M | Canada | North America | turtle | 1958 | [2] |
|  | *Entamoeba* RL6 | AF149911 | NIH:1091 | USA | North America | reptile | 1999 | [2] |
|  | *Entamoeba* RL5 | FR686365 | Oedla | Sweden | Europe | reptile | 2010 | [1] |
|  | *Entamoeba* RL10 | KRO25408 | Yin | Amsterdam | Europe | elephant | 2015 | [4] |
| Uninucleated | *E. polecki* ST2 | MH620471 | 20140619F012 | China | Asia | monkey | 2015 | [14] |
|  | *E. polecki* | LC082304 | IMN | Indonesia | Asia | monkey | 2013 | [16] |
|  | *E. polecki* ST1 | AF149913 | NIH:1293:1 | USA | North America | pig | 1997 | [2] |
|  | *E. polecki* ST4 | FR686400 | UNE9 | Sweden | Europe | human | 2001 | [1] |
|  | *E. polecki* ST3 | AJ566411 | N/A | Spain | Europe | ostrich | 2004 | [17] |
|  | *E. polecki* ST4 | FR686357 | J69 | Netherlands | Europe | human | 2011 | [1] |
|  | *E. polecki* ST2 | AF149912 | NIH:0191:1 | USA | North America | monkey | 1997 | [2] |
|  | *E. bovis* | FN666252 | Reindeer100 | Iceland | Europe | ruminant | 2010 | [18] |
|  | *E. suis* | DQ286372 | Hue | Vietnam | Asia | pig | 2003 | [15] |
|  | *Entamoeba* RL1 | FN666253 | Roedeer352 | Sweden | Europe | doe | 2010 | [18] |
|  | *Entamoeba* RL3 | FR686358 | Hulman | Germany | Europe | monkey | 2010 | [1] |
|  | *Entamoeba* RL3 | FR686359 | 09/1247 | UK | Europe | monkey | 2011 | [1] |
| Cyst data n/a | *Entamoeba* RL4 | FR686361 | C0w4 | Libya | Africa | ruminant | 2010 | [1] |
|  | *Entamoeba* RL2 | FR686363 | Cow350 | Sweden | Europe | ruminant | 2010 | [1] |
|  | *Entamoeba* RL2 | FR686362 | Cow349.2 | Sweden | Europe | ruminant | 2010 | [1] |
|  | *Entamoeba* RL11 | KR025409 | KF3 | UK | Europe | rodent | 2015 | [4] |
| Non-cyst former | *E. gingivalis* | D28490 | ATCC 30927 | N/A | N/A | human | 1999 | [19] |

N/A: data not available

**References**

1. Stensvold CR, Lebbad M, Victory EL, Verweij JJ, Tannich E, Alfellani M, et al. Increased sampling reveals novel lineages of *Entamoeba*: consequences of genetic diversity and host specificity for taxonomy and molecular detection [published correction appears in Protist. 2016 Feb;167(1):31]. Protist. 2011;162(3):525-541.
2. Silberman JD, Clark CG, Diamond LS, Sogin ML. Phylogeny of the genera *Entamoeba* and *Endolimax* as deduced from small-subunit ribosomal RNA sequences. Mol Biol Evol. 1999;16(12):1740-1751.
3. Kobayashi S, Suzuki J, Takeuchi T. Establishment of a continuous culture system for *Entamoeba muris* and analysis of the small subunit rRNA gene. Parasite. 2009;16(2):135-139.
4. Jacob AS, Busby EJ, Levy AD, Komm N, Clark CG. Expanding the *Entamoeba* Universe: New Hosts Yield Novel Ribosomal Lineages. J Eukaryot Microbiol. 2015;63(1):69-78.
5. Tachibana H, Yanagi T, Pandey K, Cheng XJ, Kobayashi S, Sherchand JB, et al. An *Entamoeba* sp. strain isolated from rhesus monkey is virulent but genetically different from *Entamoeba histolytica*. Mol Biochem Parasitol. 2007;153(2):107-114.
6. Novati S, Sironi M, Granata S, Bruno A, Gatti S, Scaglia M, et al. Direct sequencing of the PCR amplified SSU rRNA gene *of Entamoeba dispar* and the design of primers for rapid differentiation from *Entamoeba histolytica*. Parasitology. 1996;112(Pt4):363-369.
7. Al-abodi H. Molecular characterization of *Entamoeba* spp in Al-Qadisiya province, Iraq. Al-Kufa University Journal for Biology. 2018;9:374-386.
8. Chavatte JM, Jureen R. Incidental Detection of *Cyclospora cayetanensis* during General Health Screening: A Case Study from Singapore. J Trop Dis. 2016;4(5):224.
9. Suzuki J, Kobayashi S, Imada M, Tolba ME, Takeuchi T. Characterization of a novel *Entamoeba histolytica* strain from Burkina Faso, Africa, possessing a unique hexokinase-2 gene. Parasite. 2011;18(4):287-294.
10. Ramachandran S, Bhattacharya A, Bhattacharya S. Nucleotide sequence analysis of the rRNA transcription unit of a pathogenic *Entamoeba histolytica* strain HM-1:IMSS. Nucleic Acids Res. 1993;21(8):2011.
11. Sogin ML, Edman U, Elwood HE, Agabian N. Small subunit ribosomal RNA from *Entamoeba histolytica*. 1992. Unpublished. Avaible in <https://www.ncbi.nlm.nih.gov/nuccore/X56991>.
12. Guan Y, Feng M, Cai J, Min X, Zhou X, Xu Q, et al. Comparative analysis of genotypic diversity in *Entamoeba nuttalli* isolates from Tibetan macaques and rhesus macaques in China. Infection, Genetics and Evolution. 2016;38:126-131.
13. Feng M, Cai J, Min X, Fu Y, Xu Q, Tachibana H, et al. Prevalence and genetic diversity of *Entamoeba* species infecting macaques in southwest China. Parasitol Res. 2013;112(4):1529–1536.
14. Chang AM, Chen CC, Huffman MA. *Entamoeba* spp. in wild formosan rock macaques (*Macaca cyclopis*) in an area with frequent human-macaque contact. J Wildl Dis. 2019;55(3):608-618.
15. Clark CG, Kaffashian F, Tawari B, Windsor JJ, Twigg-Flesner A, Davies-Morel MCG, et al. New insights into the phylogeny of *Entamoeba* species provided by analysis of four new small-subunit rRNA genes. Int J Syst Evol Microbiol. 2006;56(Pt9):2235-2239.
16. Tuda J, Feng M, Imada M, Kobayashi S, Cheng X, Tachibana H. Identification of *Entamoeba polecki* with Unique 18S rRNA Gene sequences from Celebes Crested Macaques and Pigs in Tangkoko Nature Reserve, North Sulawesi, Indonesia. J. Eukaryot. Microbiol. 2007;63(5):572-577.
17. Ponce GF, Martínez DRA, Herrera S. *Entamoeba struthionis* n.sp. (Sarcomastigophora: Endamoebidae) from ostriches (*Struthio camelus*). Vet Parasitol. 2004;119(4):327-335
18. Stensvold CR, Lebbad M, [Clark CG](http://researchonline.lshtm.ac.uk/view/creators/ipmbgcla.html). Genetic characterisation of uninucleated cyst-producing *Entamoeba* spp. from ruminants. International journal for parasitology. 2010;40(7):775-778.
19. Yamamoto A, Kikuta N, Hashimoto T, Oyaizu H, Goto N. Nucleotide sequence of the SrRNA gene of *Entamoeba gingivalis*: applications for construction of a species-specific DNA probe and phylogenetic analysis. Microbiol Immunol. 1995;39(3):185-92.
